# CircRNAs: Pivotal modulators of TGF-β signalling in cancer pathogenesis

**DOI:** 10.1016/j.ncrna.2024.01.013

**Published:** 2024-01-26

**Authors:** Asif Ahmad Bhat, Gaurav Gupta, Rajiv Dahiya, Riya Thapa, Archana Gahtori, Moyad Shahwan, Vikas Jakhmola, Abhishek Tiwari, Mahish Kumar, Harish Dureja, Sachin Kumar Singh, Kamal Dua, Vinoth Kumarasamy, Vetriselvan Subramaniyan

**Affiliations:** a School of Pharmacy, Suresh Gyan Vihar University, Jagatpura, Mahal Road, Jaipur, India; b School of Pharmacy, Graphic Era Hill University, Dehradun, 248007, India; c Centre of Medical and Bio-allied Health Sciences Research, Ajman University, Ajman, Ajman, 346, United Arab Emirates; d School of Pharmacy, Faculty of Medical Sciences, The University of the West Indies, St. Augustine, Trinidad & Tobago; e Department of Pharmaceutical Chemistry, School of Pharmaceutical Sciences, Shri Guru Ram Rai University, Dehradun, 248001, Uttarakhand, India; f Department of Clinical Sciences, College of Pharmacy and Health Sciences, Ajman University, Ajman, 346, United Arab Emirates; g Uttaranchal Institute of Pharmaceutical Sciences, Uttaranchal University, Dehradun, 248007, India; h Pharmacy Academy, IFTM University, Lodhipur-Rajput, Moradabad, (U.P.), 244102, India; i Department of Pharmaceutics, ISF College of Pharmacy, Moga, Punjab, India; j Department of Pharmaceutical Sciences, Maharshi Dayanand University, Rohtak, Haryana, 124001, India; k School of Pharmaceutical Sciences, Lovely Professional University, Phagwara, 144411, India; l Faculty of Health, Australian Research Centre in Complementary and Integrative Medicine, University of Technology, Sydney, Ultimo, NSW, 2007, Australia; m Discipline of Pharmacy, Graduate School of Health, University of Technology, Sydney, Ultimo, NSW, 2007, Australia; n Department of Parasitology and Medical Entomology, Faculty of Medicine, Universiti Kebangsaan Malaysia, Jalan Yaacob Latif, 56000, Cheras, Kuala Lumpur, Malaysia; o Pharmacology Unit, Jeffrey Cheah School of Medicine and Health Sciences, Monash University, Jalan Lagoon Selatan, Bandar Sunway, 47500, Selangor Darul Ehsan, Malaysia

**Keywords:** circRNAs, TGF-β, Cancer, Cancer biology, Epithelial-mesenchymal transition

## Abstract

The intricate molecular landscape of cancer pathogenesis continues to captivate researchers worldwide, with Circular RNAs (circRNAs) emerging as pivotal players in the dynamic regulation of biological functions. The study investigates the elusive link between circRNAs and the Transforming Growth Factor-β (TGF-β) signalling pathway, exploring their collective influence on cancer progression and metastasis. Our comprehensive investigation begins by profiling circRNA expression patterns in diverse cancer types, revealing a repertoire of circRNAs intricately linked to the TGF-β pathway. Through integrated bioinformatics analyses and functional experiments, we elucidate the specific circRNA-mRNA interactions that modulate TGF-β signalling, unveiling the regulatory controls governing this crucial pathway. Furthermore, we provide compelling evidence of the impact of circRNA-mediated TGF-β modulation on key cellular processes, including epithelial-mesenchymal transition (EMT), migration, and cell proliferation. In addition to their mechanistic roles, circRNAs have shown promise as diagnostic and prognostic biomarkers, as well as potential molecular targets for cancer therapy. Their ability to modulate critical pathways, such as the TGF-β signalling axis, underscores their significance in cancer biology and clinical applications. The intricate interplay between circRNAs and TGF-β is dissected, uncovering novel regulatory circuits that contribute to the complexity of cancer biology. This review unravels a previously unexplored dimension of carcinogenesis, emphasizing the crucial role of circRNAs in shaping the TGF-β signalling landscape.

## 1 Introduction

Cancer, a multifaceted and complex array of diseases, continues to pose a formidable challenge to the field of medicine. As researchers strive to decipher the intricacies of its molecular landscape, the role of ncRNAs has emerged as a focal point of investigation [1]. CircRNAs have recently drawn focus for their regulatory roles in diverse cellular processes, including those implicated in cancer pathogenesis. Among the numerous signalling pathways orchestrating cellular behaviour, the TGF-β pathway stands out as a key player in carcinoma progression and metastasis [2,3]. Initially identified for its function in embryonic development and tissue homeostasis, the TGF-β signalling pathway has been linked to a dual role in cancer, functioning as a tumour promoter and a suppressor based on the disease's setting and stage [4]. Its intricate network of ligands, receptors, and downstream effectors regulates pivotal cellular processes, such as EMT, apoptosis, differentiation, and cell proliferation [5]. Dysregulation of the TGF-β pathway is a common hallmark in various cancers, contributing to tumour initiation, progression, and metastasis [6]. CircRNAs, once considered byproducts of splicing errors, have emerged as sophisticated regulators of gene expression. Their unique circular structure, resistant to exonucleases, endows them with stability and longevity, making them intriguing candidates for mediating long-term cellular responses [7].

### 1.1 Background and biological significance

CircRNAs have become important modulators of gene expression because of their ability to build a covalently closed-loop structure via back-splicing activities. Initially dismissed as splicing artifacts, the advent of high-throughput sequencing technologies has facilitated the identification and characterization of a plethora of circRNAs across various species and tissues [8]. Unlike linear RNAs, circRNAs lack 5′ caps and 3′ polyadenylated tails, rendering them resistant to degradation by exonucleases. This unique structural stability has increased interest in understanding their functional significance in cellular processes [9]. The diverse functions of circRNAs, including sequestering microRNAs (miRNAs) through competitive binding, acting as protein sponges, and influencing alternative splicing [10]. The regulatory potential of circRNAs extends to the modulation of signalling pathways, making them key players in cellular homeostasis and disease [11].

The TGF-β signalling pathway, initially identified for its role in tissue homeostasis, embryonic development, and immune regulation, has emerged as a central player in cancer biology [12]. This pathway encompasses a family of structurally related ligands, including TGF-β1, TGF-β2, and TGF-β3, which elicit signals via a receptor complex consisting of serine/threonine kinase receptors of types I (TβRI) and II (TβRII) [13]. When the ligand binds, the receptor complex initiates a cascade of intracellular events, culminating in the activation of Smad proteins, the canonical mediators of TGF-β signalling. Although TGF-β signalling suppresses tumour growth and triggers apoptosis in the first phases of carcinogenesis, its function in later cancer stages is more nuanced. TGF-β may accelerate the growth of tumours by improving invasion, metastasis, and immune evasion in their latter stages [14,15].

The intricate interplay between circRNAs and the TGF-β signalling pathway has become a focal point of investigation, as researchers seek to unravel the molecular mechanisms underlying their cooperative or antagonistic roles in cancer [16]. The regulatory crosstalk between circRNAs and TGF-β signalling is multifaceted, involving diverse mechanisms that impact various aspects of cellular behaviour. Numerous studies have demonstrated that circRNAs can modulate the expression of key components of the TGF-β signalling pathway, influencing its activation and downstream effects [17,18]. One mechanism through which circRNAs exert this regulatory function is by acting as sponges for miRNAs that target TGF-β pathway components. In addition to miRNA sponging, circRNAs can directly interact with TGF-β pathway components, modulating their stability, subcellular localization, or activity [19]. Circ-Foxo3 has been reported to bind directly to the TβRI and promote its degradation, thereby attenuating TGF-β signalling and inhibiting tumour progression. Beyond their influence on TGF-β signalling components, circRNAs actively participate in the modulation of cellular processes regulated by the TGF-β pathway [20]. One of the hallmark processes influenced by TGF-β is the EMT, a critical event in cancer metastasis. CircRNAs have been associated in the regulation of TGF-β-induced EMT, either by directly impacting EMT-related genes or by modulating manifestation of primary transcription factors involved in EMT [21]. The dysregulation of circRNAs and the TGF-β pathway in cancer has profound ramifications for therapeutic, prognostic, and diagnostic intervention. The identification of specific circRNA signatures associated with TGF-β dysregulation in patient samples holds promise for precision medicine, enabling the stratification of patients based on their molecular profiles [22,23]. This comprehensive review aims to unravel the intricate relationship between circRNAs and the TGF-β signalling pathway, focusing on their collective influence on cancer pathogenesis.

## 2 The transforming growth factor-β pathway in cancer

The TGF-β family encompasses a group of structurally related cytokines, including TGF-β3, TGF-β2, and TGF-β1, that orchestrate cellular responses through the activation of a binding complex [24]. The canonical TGF-β signalling cascade is initiated when TGF-β ligands bind to a type II (TβRII) serine/threonine kinase and heteromeric complex of type I (TβRI) receptors [25]. This ligand-receptor interaction leads to the phosphorylation of downstream effector Smad proteins, specifically Smad3 and Smad 2. To control the transcription of certain genes, phosphorylated R-Smads form complexes with the ubiquitous Smad 4, Smad, and then move into the nucleus, where they work with other co-factors [26]. The spectrum of TGF-β target genes includes those involved in cell cycle control, apoptosis, immunological response, extracellular matrix (ECM) formation, and the EMT. The TGF-β pathway acts as a tumour suppressor by regulating cell proliferation and triggering apoptosis in the early stages of carcinogenesis [27]. By downregulating c-Myc, a major promoter of cell proliferation, and upregulating cyclin-dependent kinase inhibitors like p21 and p15, TGF-β signalling suppresses the cell cycle. Simultaneously, TGF-β stimulates the expression of pro-apoptotic factors, contributing to the elimination of potentially harmful cells [28]. This tumour-suppressive phase serves as a protective mechanism against the uncontrolled proliferation of damaged cells, emphasizing the physiological significance of the TGF-β pathway in maintaining cellular homeostasis [29].

**Fig. 1:**
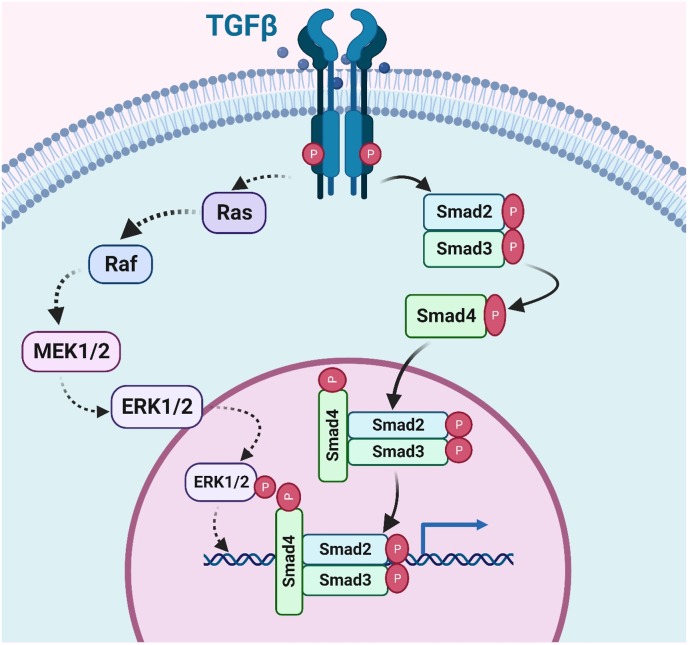
The diagram explains the complex molecular mechanisms that underlie the critical function of Transforming Growth Factor-β (TGF-β) signalling in the aetiology of cancer. It describes how TGF-β activation sets off a series of molecular events that then modify important cellular mechanisms involved in oncogenesis.

Through a process of EMT, caused by TGF-β, cancer cells can more easily migrate and invade into other tissues. During EMT, cells acquire enhanced motility and invasive properties, allowing them to breach tissue boundaries and initiate metastasis [30]. Moreover, TGF-β exerts immunosuppressive effects by inhibiting the activity of cytotoxic T cells and natural killer (NK) cells, fostering an immune-tolerant microenvironment conducive to tumour survival and progression. The dichotomous role of the TGF-β pathway in cancer has significant clinical implications, shaping the development of therapeutic strategies [31]. While attempts to globally target the TGF-β pathway have faced challenges due to its dual nature, precision medicine approaches aim to modulate its activity based on the specific context of the tumour. In cancers characterized by intact tumour-suppressive TGF-β signalling, therapeutic strategies focus on restoring its function [32]. This may involve the development of agonists that enhance TGF-β-mediated growth inhibition and apoptosis, providing a targeted approach for cancers in which the pathway remains dormant [33]. Conversely, in tumours where the TGF-β pathway promotes tumorigenesis, therapeutic efforts concentrate on inhibiting its pro-tumorigenic effects [34]. Small-molecule inhibitors targeting key components of the TGF-β signalling cascade, such as TβRI kinase inhibitors, have shown promise in preclinical studies and early-phase clinical trials, offering a potential avenue for disrupting the pro-tumorigenic phase of TGF-β signalling [35,36] (Fig. 1).

## 3 Interplay between circular RNAs and the transforming growth factor-β

The intricate web of molecular interactions that govern cellular processes in health and disease has expanded to include circRNAs as essential players in the regulatory landscape [37]. In recent times, a growing research evidence has highlighted the interplay between circRNAs and the TGF-β signalling pathway, revealing a complex network of interactions that contribute to the modulation of cellular responses in various physiological and pathological contexts [38]. The crosstalk between circRNAs and the TGF-β pathway has emerged as a fascinating area of research, uncovering intricate regulatory mechanisms that influence cellular responses. One prominent mode of interaction involves circRNAs acting as miRNA sponges, thereby sequestering miRNAs that target key components of the TGF-β signalling cascade [39,40]. circRNA-ITCH has been identified as a potent sponge for miR-17 and miR-224. By binding to these miRNAs, circRNA-ITCH relieves their inhibitory effects on TGF-β1 and Smad7, contributing to elevated TGF-β signal transduction [41]. This regulatory axis illustrates how circRNAs can fine-tune the amplitude and duration of TGF-β signalling, impacting downstream cellular processes such as EMT and apoptosis. Beyond miRNA sponging, circRNAs exert their influence on the TGF-β pathway through direct interactions with its key components [42]. Circ-Foxo3, for example, has been reported to bind directly to the TβRI receptor, promoting its degradation and attenuating TGF-β signalling. This mechanism highlights the diversity of strategies employed by circRNAs to modulate the pathway, contributing to the nuanced regulation of cellular responses [43]. The interplay between circRNAs and TGF-β signalling extends its influence to critical cellular processes, with profound implications for cancer and other diseases. The TGF-β-induced EMT and other processes may be regulated by circRNAs. Through their intricate modulation of TGF-β signalling, circRNAs can influence the phenotypic plasticity of cells, impacting migration, invasion, and metastatic potential [44]. Moreover, the dysregulation of circRNAs in TGF-β signalling has been associated with various pathologies, underscoring their clinical relevance [45,46]. Altered expression profiles of circRNAs in cancer tissues and biofluids offer potential diagnostic and prognostic markers, providing valuable insights into the dynamic interplay between circRNAs and TGF-β signalling in disease progression [47]. This interplay not only expands our understanding of the complexity of gene regulation but also unveils novel therapeutic targets and diagnostic biomarkers for diseases, particularly cancer [48]. As researchers delve deeper into this intricate interplay, the potential for clinical translation and transformative advances in personalized medicine beckons, promising a future where the manipulation of circRNAs in TGF-β signalling contributes to more effective and tailored therapeutic interventions [49].

## 4 Functional implications of LncRNA through Hedgehog signaling pathway in cancer

### 4.1 Breast cancer

Breast cancer is the most common malignant neoplastic disease and the second leading cause of death worldwide. This deleterious condition originates from unbridled cellular proliferation within mammary tissue [50]. Marked by intricate genomic modifications, breast carcinoma epitomizes a polymorphic disorder encompassing diverse molecular subtypes. The progression of this disease is intricately modulated by a spectrum of determinants, including hormonal, genetic, and environmental influences, thereby unfolding across varied developmental stages [51,52]. The Apelin signalling pathway, a crucial regulatory system in physiology, centers around the Apelin peptide and its cognate G protein-coupled receptor. Implicated in cardiovascular homeostasis, energy metabolism, and neuroendocrine regulation, Apelin signalling exhibits diverse effects in various tissues [53]. As a key modulator of vascular function and cardiac contractility, understanding the intricacies of the Apelin pathway holds promise for therapeutic interventions in cardiovascular diseases and metabolic disorders [54]. Lin et al. established a ceRNA regulation network by identifying 8 DEcircRNAs, 25 miRNAs, and 216 mRNAs. Apelin signalling pathway, TGF-β, and transcription factor binding were highly enriched. In addition to being favourably connected to cell cycle and proliferation, increased hub genes (KPNA2 and RACGAP1) were also associated with a worse prognosis. A sub-network for prognosis was built with 6 nodes: 2 circRNAs, 4 miRNAs, and 2 mRNAs. Researchers found that silencing circ_0001583 and circ_0008812 had a profound effect in preventing the growth of MCF-7 cells [55]. circRNAs, lncRNAs, and mRNAs all play important roles in the ceRNA regulation network. These RNA species compete for common miRNAs, serving as molecular sponges to control the expression of one another in this complex network [56]. The dynamic and interrelated nature of the RNA world inside cells has been brought to light by the ceRNA idea, which has greatly advanced our knowledge of post-transcriptional gene regulation. There are several physiological and pathological states that may be affected by changes in this regulatory network [57]. Wang et al. developed a ceRNA regulation network after identifying 144 differentially expressed DEcircRNA, 221 DEmiRNA, and 1211 DEmRNA. There was a total of 78 mRNA, 42 miRNAs, and 42 circRNAs in the network. Genes with high levels of receptor activity triggered by TGF-beta and epithelial cell death were found. There are four genes that have been linked to breast cancer survival and prognosis [58].

### 4.2 Colorectal cancer

Colorectal cancer (CRC) stands as a prominent contributor to global cancer-related mortality. The progression of CRC transpires from benign polyps to aggressive carcinomas, traversing sequential phases marked by an accumulation of genetic aberrations and epigenetic modifications [59]. Age, familial predisposition, and lifestyle choices emerge as potential etiological factors. Prognosis significantly benefits from early detection through screening modalities [60]. Therapeutic modalities, encompassing surgical interventions, chemotherapeutic regimens, and immunotherapeutic approaches, aim to eradicate or control the neoplastic process [61]. Despite advancements, the persistent prominence of CRC in cancer-related morbidity and mortality underscores the ongoing importance of preventive initiatives and therapeutic advancements [62,63]. During embryonic development and subsequent B-cell differentiation, PAX5 plays a vital function as a crucial transcription factor. The protein encoded by the PAX5 gene coordinates critical molecular processes, such as the expression of genes required specifically for the growth and maintenance of B lymphocytes [64]. In addition to its function throughout development, PAX5 has been linked to a wide range of haematological cancers due to the role its dysregulation plays in driving uncontrolled B-cell proliferation. PAX5 is essential for various pathological and physiological processes, but its complexity highlights its importance in the setting of cancer [65]. Yu et al. examined the expression levels of circRNA in primary CRC tissues, surrounding normal tissues, and CRC tissues that had metastasized to the liver. In CRC patients with liver metastases, circumRNA hsa_circ_0020134 (circ0020134) was found to be increased, suggesting a bad prognosis. In vitro and in vivo, Circum 0020134 stimulates the growth and spread of colorectal cancer cells. PAX5 induces the upregulation of circ0020134, whilst miR-183–5p functions as a sponge. EMT in CRC cells is inhibited by downregulation and may be reverted with treatment with miR-183–5p inhibitors [66]. An essential nervous system route called neurotrophin signalling controls the growth, survival, and plasticity of neurons. When neurotrophins, like nerve growth factor (NGF) and brain-derived neurotrophic factor (BDNF), attach to their receptors, a series of intracellular processes are set off [67]. Neuronal function is modulated by activation of Trk receptors and p75NTR, which affects synaptic plasticity and cellular survival. Gaining knowledge about neurotrophin signalling may help treat neurological illnesses and give insights into how neurons evolve [67]. Important circRNAs connected to the development of colon cancer were discovered by Yang et al. High-throughput RNA sequencing was used to both normal and colon tumour samples. mRNAs and circRNAs with differential expression were found; 408, 472, and 278 circRNAs with differential expression were found. Circulations 052666, 022743, and 004452 were found to be enriched in the extracellular matrix/receptor interaction, neurotrophin signalling route, and TGF-β signalling pathway, respectively, according to functional enrichment analysis [68]. The characteristic of stem cells known as stemness is controlled by complex signalling pathways that control differentiation and self-renewal. Wnt, Notch, Hedgehog, and TGF-β are among the stemness-associated pathways that work together to maintain pluripotency and the potential to generate a variety of cell lineages [69]. Understanding the molecular subtleties of these pathways is crucial for maintaining tissue homeostasis and cellular growth. It also has great potential for regenerative medicine and cancer treatments [70]. The regulatory networks of CSC-enriched CRC spheroid cells were mapped by Rengganaten et al., who also found a key network of mRNA molecules that modulate stemness-associated signalling pathways. In the spheroid cells, the expression levels of two significant circRNAs, hsa_circ_0082096 and hsa_circ_0066631, were up-regulated, whereas miR-224, miR-382, miR-548c-3p, miR-579, and miR-140–3p, were down-regulated. Six mRNA targets were blocked by these circRNAs, regulating different facets of CSC stemness [71].

### 4.3 Pancreatic cancer

**Fig. 2:**
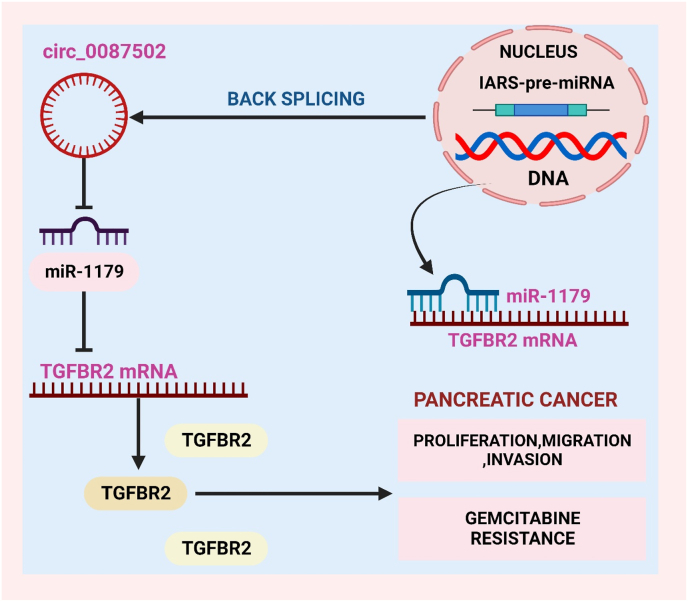
This image illustrates the pivotal role of circular RNAs, circ_0087502, in human carcinogenesis, metastasis, and chemoresistance. Elevated expression of circ_0087502 in pancreatic cancer tissues and cell lines signifies a poor prognosis. The image illustrates reduced cell proliferation, migration, and invasion, enhancing the efficacy of gemcitabine in combating pancreatic cancer.

Pancreatic cancer poses a global health threat due to its aggressive nature and often late detection. Infamous for its slow progression and challenges in early identification, this cancer frequently eludes timely diagnosis [72]. Most cases are discovered at advanced stages, restricting treatment options, and resulting in a grim prognosis. Risk factors encompass age, smoking, family history, and genetics. The subtle symptoms further complicate diagnosis. Conventional treatments such as surgery, chemotherapy, and radiation prove arduous [73]. However, ongoing research into the molecular complexity of pancreatic cancer holds promise for personalized therapies. Emerging techniques aim to enhance early detection and develop more efficacious treatments, instilling hope in the battle against this formidable disease [74,75]. Chen et al. found that Circular RNAs like circ_0087502 are essential to human carcinogenesis, metastasis, and chemoresistance. Compared to normal cells, pancreatic cancer tissues and cell lines express more circ_0087502, which worsens prognosis. It decreased cell proliferation, migration, and invasion, making gemcitabine more effective as shown in Fig. 2 [76]. In the complex world of gene regulation, circular RNAs (circRNAs) like circEIF3I are crucial. Back-splicing of the EIF3I gene creates a closed loop, referred to as circEIF3I. Stable and resistant to exonucleolytic degradation, circEIF3I may regulate [77]. Emerging research shows that circEIF3I modulates cellular processes including transcriptional control and protein translation. The regulatory activities of circEIF3I may provide new gene expression regulation and cellular homeostasis insights [78]. Using circRNAs from normal and PDAC tissues, Zhao et al. validated circEIF3I's loop structure. By upregulating MMPs, circEIF3I increased PDAC cell motility, invasion, and metastasis in vivo and in vitro. By binding to the MH2 domain, CirceEIF3I facilitates SMAD3's interactions with TGFRI on early endosomes, hence increasing SMAD3 phosphorylation. In early endosomes, AP2A1 binds with circEIF3I to directly induce SMAD3 recruitment to TGFRI [79].

### 4.4 Gastric cancer

Gastric carcinoma, originating in the gastric mucosa, constitutes a formidable global health exigency. Frequently diagnosed at advanced stages, risk factors encompass Helicobacter pylori infection, dietary variables, and genetic predisposition [80]. Stratified into intestinal and diffuse histological subtypes, gastric carcinoma commonly manifests inconspicuous symptoms, contributing to protracted latency in detection [81,82]. Surgical excision remains a pivotal therapeutic intervention, complemented by adjunctive chemotherapy and precision-targeted modalities. Despite incremental strides, the prognostic outlook remains guarded, underscoring imperatives for the development of early detection methodologies [83]. Ongoing investigative endeavors delve into elucidating the molecular underpinnings, thereby delineating prospective therapeutic targets. Preventive initiatives pivot on H. pylori eradication protocols and lifestyle modifications [84]. The intricate nature of gastric carcinoma necessitates a multidisciplinary paradigm, fostering enhanced comprehension, diagnostic precision, and therapeutic modalities. One important intracellular signalling cascade that controls cellular functions including cell growth, survival, and metabolism is the PI3K/AKT pathway [85]. Key cellular processes are controlled by AKT, which is phosphorylated and activated after PI3K activation. AKT then modifies downstream effectors. PI3K/AKT pathway dysregulation is often linked to several illnesses, including cancer, which makes it a popular target for therapeutic treatments and a main area of focus for molecular biology and medical research [86]. Li et al. discovered that gastric cancer cell lines and tissues have increased levels of both C-E-Cad and circ-E-Cad. Gastric cancer cell line growth and metastasis were inhibited by circ-E-Cad knockdown, while overexpression had the reverse effect. C-E-Cad controlled the PI3K/AKT pathway to promote tumour growth. Through the TGF-β/Smad pathway, increased expression of C-E-Cad might control gastric cancer cell motility, proliferation, and EMT [87].

**Fig. 3:**
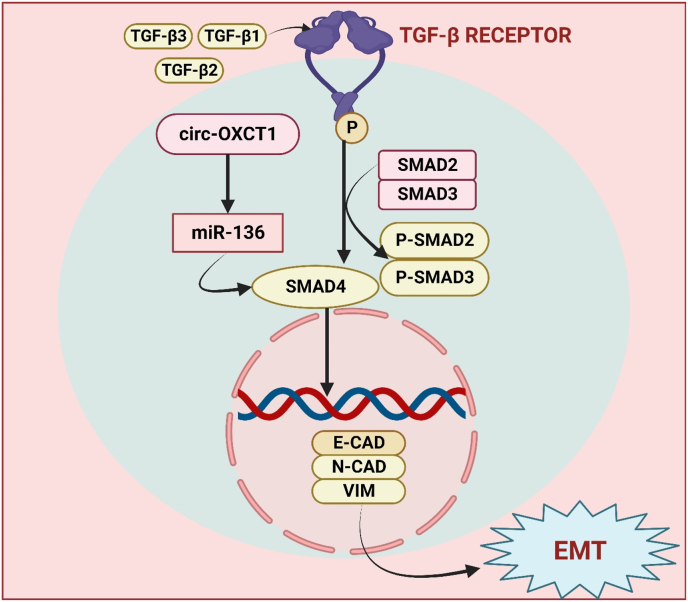
The image depicts the pivotal role of Circ-OXCT1 in inhibiting gastric cancer EMT and metastasis by modulating the TGF-β pathway through the Circ-OXCT1/miR-136/SMAD4 axis. The overexpression of Circ-OXCT1 effectively restrains cell migration and invasion, while proliferation remains unaffected. Circ-OXCT1 downregulates SMAD4 expression and suppresses EMT in gastric cancer cells through the intricacies of the circ-OXCT1/miR-136/SMAD4 axis.

A dynamic aspect of non-coding RNA biology is shown by Circ-OXCT1, a circRNA involved in a variety of cellular functions. With its covalently closed-loop structure, Circ-OXCT1 has emerged as a major participant in gene regulation from biogenesis to therapeutics [88]. It may be involved in regulating the expression of genes linked to metabolic pathways and cellular homeostasis. The regulatory aspects of this circular RNA have attracted interest, and current research aims to elucidate its precise actions and consequences in several biological situations [89,90]. Circ-OXCT1 was discovered by Liu et al., along with its role on EMT in GC. The gastric cancer tissues and cell lines exhibited downregulated circ-OXCT1, which was shown to be substantially correlated with lymph node metastasis, pathologic stage, and overall survival rate. The expression of SMAD4 was downregulated by Circ-OXCT1 silencing, which in turn controlled the expression of vimentin, N-cadherin, and E-cadherin via the TGF-β/Smad signalling pathway. In addition to increasing cell migration, invasion, and lung metastasis in naked mice, this improved EMT. Because it targets the circ-OXCT1/miR-136/SMAD4 axis, circ-OXCT1 overexpression may be a potential therapy for advanced gastric cancer, particularly when there are distant metastases as shown in Fig. 3 [91].

A microRNA known as miR-361–3p has gained notice for its regulatory functions in a variety of biological processes. MiR-361–3p has emerged as a powerful regulator of gene expression and is associated with important pathways controlling migration, apoptosis, and cell proliferation [92]. Its dysregulation is linked to several illnesses, such as cancer, highlighting its importance as a possible target for diagnosis and treatment. This little RNA molecule plays a complex role in the complex web of biological processes, advancing our knowledge of how genes are regulated in both healthy and disease states [93]. Zhou et al. discovered that circ_0006089 was highly expressed in GC tissues and cells, where it served as a sponge for miR-361–3p. Reducing circ_0006089 levels inhibited GC growth, spread, metabolism, angiogenesis, and apoptosis. The detrimental effects of miR-361–3p on GC cell activity were counteracted when circ_0006089 was overexpressed. Inhibition of GC carcinogenesis when circ_0006089 was silenced was mediated via the miR-361–3p/TGFB1 pathway, indicating that circ_0006089 might be a useful therapeutic target for GC [94].

A vital part of the cell's defence systems, the p53 signalling pathway controls cell division, DNA repair, and apoptosis. The p53, is a tumour suppressor protein that is turned on in response to cellular stress [95]. Its complex signalling cascade coordinates a cell's reaction to threats to its genome and stops damaged cells from multiplying. The relationship between the p53 pathway's deregulation and certain cancer types highlights the pathway's critical role in preserving cellular homeostasis [96,97]. The Oe-circ_0067582 group reduced cell viability, proliferation, and invasion capacity, and promoted apoptosis in AGS and SGC-7901 cells, as shown by research by Lu et al. SGC-7901 tumour-bearing nude mice had their tumour development slowed because of an increase in cysteinyl aspartate specific proteinase 3 protein levels. Cancer-related biological processes that miRNA targets included suppression of apoptosis, gene expression, transcriptional misregulation, transforming growth factor, and p53 signalling [98]. The circular RNA (circRNA) CircCCDC66 has attracted interest because of its possible function as a regulator of cellular activities. A closed-loop structure emerges from the CCDC66 gene, setting it apart from its linear RNA homologues [99]. Exploring the roles and interactions of CircCCDC66 gives vital insights into the complex world of non-coding RNAs and their influence on cellular physiology and disease, since circRNAs are becoming more recognised as key participants in gene expression control [100]. The function of circCCDC66 in stomach cancer and its causes were studied by xu et al. Upregulation of circCCDC66 expression was shown to correlate with tumour grade and lymphatic metastasis in this investigation of gastric cancer cell lines and tissues. GC cell proliferation, migration, and invasion were all considerably reduced, cell apoptosis was triggered, and carcinogenesis was prevented in nude mice when CircCCDC66 was knocked down. In addition to correcting EMT in GC cells, reduction of circCCDC66 also reduced signalling via c-Myc and TGF-β [101].

### 4.5 Lung cancer

Lung neoplasia represents a highly deleterious oncogenic phenomenon stemming from dysregulated cellular proliferation within pulmonary tissues, significantly contributing to an elevated global morbidity and mortality attributed to neoplastic pathologies [102]. Etiological determinants encompass multifaceted variables such as tobacco exposure, environmental carcinogens, and hereditary predispositions. Categorically, lung cancer is dichotomized into Non-Small Cell Lung Cancer (NSCLC) and Small Cell Lung Cancer (SCLC) [103,104]. NSCLC, prevailing as the predominant subtype, is renowned for its proclivity to present belatedly, in stark contrast to the more aggressive SCLC, which frequently manifests in advanced stages. Emphasizing the imperative of early surveillance for individuals at heightened risk is paramount, given the potential for asymptomatic incipient-stage lung malignancies [105]. Therapeutic modalities encompass a spectrum of interventions, including surgical interventions, chemotherapy regimens, radiation protocols, and avant-garde targeted therapeutics and immunomodulatory approaches [106,107]. Despite discernible advancements in therapeutic strategies, the prognostic landscape for lung cancer remains ominous, underscoring the exigency for sustained investigative pursuits, early detection methodologies, and innovative pharmacotherapeutic agents to efficaciously navigate its clinical trajectory [108]. In cellular biology, the Bcl-3 axis is an essential signalling route that controls important functions including inflammation, immunological response, and cell survival [109,110]. Bcl-3 is a member of the IB family and plays a crucial role in regulating gene expression as a transcriptional co-regulator. Its relevance in both physiological and pathological states is borne out by its dynamic interaction with different signalling pathways, especially those associated with NF–B [111]. Targets for therapeutic intervention in a wide range of illnesses, including cancer and inflammatory disorders, may be gleaned from a deeper understanding of the Bcl-3 axis [112]. Ge et al. evaluated circRNAs in NSCLC to learn more about their roles in this disease. Using a circRNA microarray, we observed that hsa_circRNA_0088036 was overexpressed in NSCLC tissue samples and cell lines. Researchers showed that knocking down hsa_circ_0088036 reduced the migration and invasion, proliferation of NSCLC cells and proteins involved in the EMT. It also accelerated NSCLC advancement by stimulating the TGFβ/Smad3/EMT signalling cascade via the miR-1343–3p/Bcl-3 axis [113].

Cellular responses to TGF-β signalling rely heavily on the TGF-β-activated kinase 1 (TAK1)-binding protein 2 (TAB2) pathway. TAB2 is an adaptor protein that connects TGF- receptors to the central kinase TAK1 [114,115]. Different biological functions, including cell proliferation, differentiation, and immunological responses, are affected by the downstream signalling cascades activated by this interaction. The TAB2 pathway is critical for the intricate regulation of TGF-β signalling and has a wide range of physiological and pathological applications [116]. Lung cancer cells proliferate and migrate more easily when circ-WHSC1 is present, as discovered by Guan et al. It also controls TAB2 expression by acting as a sponge for micro-RNA-7. Inhibiting the circ-WHSC1/miR-7/TAB2 pathway might significantly attenuate lung cancer development, validating its oncogenic role in NSCLC and recommending it as a possible target for NSCLC treatment [117]. Important cellular activities including proliferation, differentiation, and stimulus response are all governed by the MAPK signalling cascade [118]. This pathway integrates multiple signals, including growth factors and stress stimuli, to control gene expression and cellular behaviour via a cascade of protein kinases that includes p38 MAPKs, c-Jun N-terminal kinases (JNKs), and extracellular signal-regulated kinases (ERKs) [119]. Because of its central function in coordinating cellular responses, the MAPK pathway is an appealing target for therapeutic treatments, and its dysregulation is linked to several illnesses, including cancer [120,121]. Using bioinformatics research, Liang et al. aimed to determine the roles hitherto unknown circRNAs play in lung cancer. Five circRNAs with differential expression were found by analysing data from three separate Gene Expression Omnibus datasets. Two circRNAs, hsa_circ_0008274 and hsa_circ_0072088, were selected for further study. Cellular response to TGF-β stimulation, EMT, MAPK signalling pathway, and PI3K-AKT signalling pathway were among the important phrases uncovered by the functional analysis of the ceRNA network. In addition, the network foretold of crucial regulatory axes between circRNA, miRNA, and mRNA in relation to cancer development [122]. One circRNA that is essential for controlling alternative splicing and gene expression is called circular RNA epithelial splicing regulatory protein 1, or cESRP1. cESRP1, which is derived from the epithelial splicing regulatory protein 1 (ESRP1) gene, has been linked to several biological processes, including the regulation of the EMT and the advancement of cancer [123]. cESRP1 have distinct structural stability, which affects how well they operate as regulatory molecules. Its importance in forming the complicated landscape of cellular signalling and gene regulation is highlighted by its complex interactions with RNA-binding proteins and microRNAs [124]. Huang et al. discovered that chemoresistant cells had considerably lower levels of cESRP1 expression, which improved drug sensitivity by suppressing miR-93–5p. By directly binding to miR-93–5p, cytoplasmic cESRP1 may impede repression and upregulate downstream targets. Chemotherapy-responsiveness of tumours was changed by TGF-β pathway overexpression and inhibition. This implies that in individuals with SCLC, cESRP1 may function as a useful predictive biomarker and possible treatment target [125].

### 4.6 Hepatocellular carcinoma

**Fig. 4:**
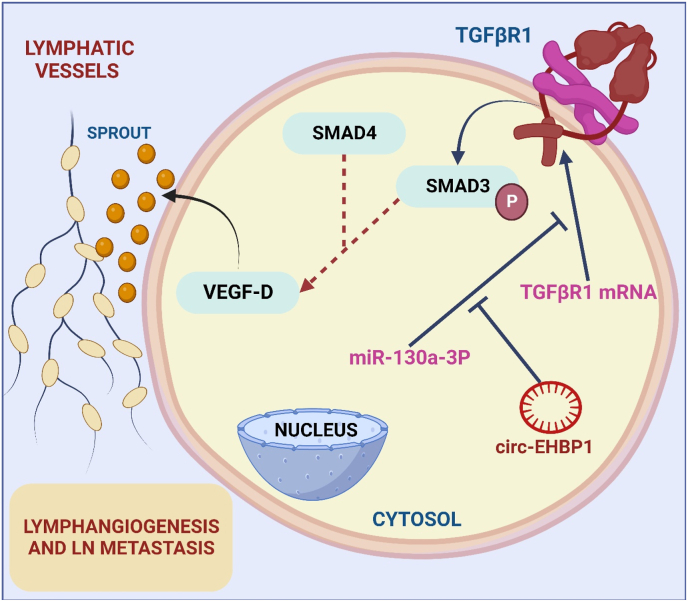
This image shows the intricate interplay between circular RNA (circEHBP1), transforming growth factor beta receptor 1 (TGFBR1), and the TGF-β/SMAD3 signalling pathway in bladder cancer. Elevated circEHBP1 levels correlate with lymphatic metastases and a poor prognosis. The cascade involves circEHBP1 upregulating TGFBR1, activating TGF-β/SMAD3, and promoting lymphangiogenesis and metastasis.

Hepatocellular carcinoma (HCC), preeminent among hepatic malignancies, constitutes a substantial global health burden [126]. Aflatoxin exposure, alcohol dependence, chronic viral hepatitis, and Nonalcoholic Steatohepatitis (NASH) emerge as pivotal etiological factors [127]. The intricacies in early HCC detection are often compounded by its intricately intertwined manifestation with cirrhosis. Therapeutic modalities predominantly encompass locoregional interventions, transplantation, and surgical resection [128,129]. Nonetheless, due to delayed disease presentations and limited therapeutic modalities, prognostication remains a formidable challenge. Although strides in systemic therapies, exemplified by sorafenib, have ameliorated outcomes, the attainment of viable curative strategies remains elusive [130]. Deeper comprehension of molecular pathways holds promise in crafting precision medications, thereby enhancing preventive strategies, early diagnostic capabilities, and therapeutic interventions for this relentless malignancy [131]. Regional RNA A special member of the circular RNA family, circFGGY is distinguished by its closed-loop structure. CircFGGY has become a prominent participant in gene regulation and has attracted interest because to its possible functions in several cellular processes [132]. Its characteristic circular shape provides durability and resistance to deterioration, which prolongs the effect on cells. Studies on circFGGY investigate its interactions with proteins and microRNAs, providing insight into its complex role in a range of physiological and pathological settings [133]. In the dynamic field of circular RNA biology, understanding the functional importance of circFGGY offers promise for the discovery of new regulatory mechanisms and possible therapeutic uses [134]. HCC growth and progression are significantly influenced by circFGGY according to Feng et al. Following hepatectomy, patients with low circFGGY expression had a low overall survival rate. It was shown that circFGGY is considerably downregulated in tumours compared to normal liver tissues. Additionally, circFGGY suppresses HCC invasion, proliferation, and epithelial-mesenchymal transition by upregulating the expression of Smad7, a gatekeeper of the TGF-β signalling pathway as shown in Fig. 4 [135].

### 4.7 Oesophageal squamous cell carcinoma

Oesophageal squamous cell carcinoma (ESCC) constitutes a prevailing and aggressive malignancy originating from the epithelial lining of the oesophagus [136]. Its pathogenesis is intricately associated with a numerous of risk factors, encompassing alcohol consumption, tobacco use, dietary insufficiencies, and chronic mucosal irritation [137]. Frequently manifesting at advanced stages, ESCC exhibits a heightened mortality rate. Its geographical predilection underscores a multifaceted etiological framework. Diagnostic modalities include endoscopic examination and histopathological biopsy [138,139]. Therapeutic interventions for ESCC entail surgical procedures, chemotherapy regimens, and radiation therapy, with prognostic implications contingent upon the disease's stage at the time of clinical manifestation [140]. Molecular profiling initiatives have discerned potential therapeutic targets, facilitating the evolution of personalized treatment modalities. Despite these strides, ESCC remains formidable due to its intrinsic heterogeneity and proclivity for late-stage diagnosis [141]. This underscores the imperative for sustained research endeavors aimed at refining early detection methodologies and devising precisely targeted therapeutic strategies to augment patient outcomes [142]. The DOCK5 gene produces the circular RNA Circ-DOCK5, which has become a prominent participant in the complex world of non-coding RNAs. Circ-DOCK5 has a stable closed-loop structure that makes it resistant to exonucleases, which makes it a possible regulator of many different cellular processes [143]. Due to its complex connections, circ-DOCK5 is becoming a more interesting topic for study, especially when it comes to cancer and other disorders. The context for examining the many functions and significance of circ-DOCK5 in cellular physiology and disease is established by this introduction [144]. Meng et al. discovered circ-DOCK5, a new circular RNA that is directly controlled by eIF4A3 and ZEB1. In tissues from ESCC, it was shown to be downregulated, which was associated with a worse prognosis. ZEB1-enhanced migration and invasion in ESCC were partly mitigated by circ-DOCK5 by making miR-627–3p more stable. ZEB1-mediated downregulation of circ-DOCK5 further enabled this downregulation of ZEB1 and inhibition of TGF-β-induced EMT. By focusing on this signalling route, ESCC development may be inhibited [145].

### 4.8 Bladder cancer

Cells lining the bladder develop out of control, leading to the common cancer that affects people all around the world [146]. It is the sixth most prevalent malignancy and presents a significant health risk, especially to the elderly. Use of tobacco products, exposure to certain chemicals, and persistent inflammation of the bladder are recognised risk factors [147,148]. Urgency, pelvic discomfort, and haematuria are common signs of bladder cancer, making early identification essential for the best possible prognosis [149]. The illness may present in a variety of ways, from non-invasive to invasive, which affects how it is treated. Advancements in the fields of diagnosis and treatment, such as immunotherapy, targeted medicines, and surgery, highlight the continuous endeavours to improve the management of bladder cancer [150]. Progressing preventive and treatment paradigms for this common and possibly fatal illness requires a thorough grasp of its aetiology, molecular complexities, and available therapies [151]. CirRIP2 signifies a growing area of study with broad ramifications. Due to its essential function in facilitating interactions with circRNAs, this circRNA Interacting Protein 2 is gaining notice. CirRIP2, a pivotal player in circRNA synthesis and function, is well-positioned to impact the control of gene expression, cell signalling pathways, and several cellular functions [152]. Cracking the code of CirRIP2's molecular connections might lead to new discoveries about the wider field of cellular physiology and disease, as well as a greater comprehension of circRNA dynamics [153]. CirRIP2 was shown to be involved in bladder cancer via generating EMT, according to research by Su et al. Higher levels of circRIP2 expression in patients were linked to the grade, stage, metastasis, and prognosis of bladder cancer. In bladder cancer, blocking TGF-β2 stopped EMT and circRIP2-induced cancer growth. This implies that circRIP2 may be a viable biomarker and therapeutic target for people with bladder cancer [154].

The circular RNA CircEHBP1, which is derived from the EHBP1 gene, has attracted interest as a major regulator of cells. A special back-splicing procedure results in the formation of this circular RNA, which has a closed-loop structure [155]. CircEHBP1 has been linked to many biological functions, including signalling pathways and gene expression. Its regulatory functions include controlling apoptosis, migration, and proliferation [156]. Circular RNA (circRNA) was discovered by Zhu et al. to promote lymphangiogenesis in a way that is independent of VEGF-C. In bladder cancer, there was an upregulation of circular RNA (circEHBP1), which was positively linked with lymphatic metastases and a poor prognosis. The expression of transforming growth factor beta receptor 1 (TGFBR1) was elevated by circular RNA, which in turn activated the TGF-β/SMAD3 signalling pathway, causing lymphangiogenesis and metastasis. CircEHBP1-induced lymphangiogenesis and metastases were inhibited in vivo by neutralising antibodies against VEGF-D. This shows that circEHBP1 may be a biomarker and therapeutic target for bladder cancer lymphatic metastases as shown in Fig. 4 [157]. The gene ILF3 genes for the protein known as interleukin enhancer-binding factor 3, which is involved in the processing and stability of RNA. ILF3's significance in the advancement of cancer has been the subject of several investigations [158]. In ESCC, MALR increases the stability of ILF3 mRNA, which in turn promotes the stability of HIF1α mRNA and the growth of the tumour. The RNA binding protein POP7 regulates the stability and expression of ILF3 mRNA to promote tumour growth and metastasis in breast cancer [159]. Via its modulation of ILF3's association with CDK4 mRNA, the circACTA2 promotes Ang II-induced senescence in vascular smooth muscle cells [160]. Li et al., discovered and verified circSLC38A1 in cell lines and clinical samples. By regulating ubiquitination, circSLC38A1 stabilised ILF3 protein. Integration of RNA-seq data and CUT&Tag-seq identified TGF-β2 as the circSLC38A1-ILF3 complex's functional target. Increased m6A methylation in circSLC38A1 upregulated it. CirculSLC38A1 in serum exosomes accurately diagnosed BC patients [161].

### 4.9 Prostate cancer

The prostate gland is a walnut-sized organ that is essential to male reproductive function. One of the most prevalent malignancies that affect men globally is prostate cancer [162]. Prostate cancer is characterized by aberrant cell proliferation and often manifests slowly at first. It is critical to understand the genesis, development, and management of this condition since it is a primary source of cancer-related morbidity and death [163]. The recognised risk factors of age, family history, and ethnicity highlight the need of routine screenings for early diagnosis, such as prostate-specific antigen (PSA) testing. For low-risk instances, treatment options include active surveillance; for more advanced cases, options include hormone therapy, radiation therapy, and surgery [164]. Prostate cancer treatment is changing because of research and personalized medicine advancements, which might lead to better prognoses and higher quality of life for those with the disease. The prostate gland's aberrant cell proliferation is the cause of prostate cancer (PCa), a common disease that affects men [165]. With localised tumours to advanced metastatic disease, PCa is the second most prevalent cancer in males. It presents with a variety of clinical manifestations. Prostate cancer patients may better manage their condition and achieve better results if they get early diagnosis via screening and new treatment choices [166]. According to Lv et al., the expression of hsa_circ_0063329 is significantly diminished in prostate cancer (PCa), and it achieves this effect by regulating the miR-605–5p/TGIF2 axis, slows PCa cell growth. While hsa_circ_0063329 silencing has the opposite consequences, overexpression slows PCa cell growth. An effective treatment plan for aggressive PCa may include hsa_circ_0063329 targeting [167]. A class of signalling proteins called interferons is essential to the immune system's reaction to viral infections. They are part of the antiviral defence system and are generated by cells in reaction to pathogens, especially viruses [168]. Interferons play a critical role in the body's defence against infectious threats by enhancing immune cell activity, regulating immunological responses, and preventing viral reproduction [169]. EMT was examined in prostate cancer development and metastasis by Yan et al. The study demonstrated that IFN-γ triggered EMT in PC-3M IE8 cells. In cells treated with or without IFN-γ, high-throughput sequencing was employed to test for differentially expressed miRNAs and circRNAs. We identified EMT-related circRNAs and miRNAs using qPCR. Western blot analysis showed downregulated EMT markers and increased Twist proteins. Evidence suggests that IFN-γ may induce EMT in PC-3M IE8 cells by promoting their migration and invasion. The research discovered that hsa_circ_0001085 and hsa_circ_0001165 contribute to TNF expression regulation and indirectly impact TGF-β and PI3K-AKT signalling pathways [170] (Table 1).

**Table 1:** This table summarizes the exploration of Circular RNAs in modulating the TGF-β in cancer. Key findings, biological activity, and methodology are condensed to provide a concise overview of the intricate role lncRNAs play in modulating this critical pathway in cancer development.

Cancer Type	Circular RNA	Findings	Mechanism/Biological Activity	Methodology
Breast Cancer	circ_0001583, circ_0008812	Hub genes worsen prognosis; Silencing inhibits MCF-7 growth	Identification, Silencing	Experimental analysis
	Not specified	Genes linked to survival and prognosis	Identification	High-throughput sequencing
Colorectal Cancer	circ0020134	Circ0020134 linked to bad prognosis; PAX5 regulation	Prognosis, PAX5 regulation	Tissue expression, In vitro/in vivo
	Not specified	Differentially expressed circRNAs; enriched pathways	Identification, Enrichment	High-throughput RNA sequencing
	hsa_circ_0082096, hsa_circ_0066631	Up-regulated circRNAs; blockage of CSC stemness	CSC stemness regulation	Expression analysis, Functional
Pancreatic Cancer	circ_0087502	Essential for carcinogenesis, metastasis, chemoresistance	Carcinogenesis, Metastasis, Chemoresistance	Expression analysis, Functional
	circEIF3I	Enhances PDAC cell motility, invasion, metastasis	PDAC progression, TGFβRI interaction	In vitro and in vivo studies
Gastric Cancer	circ-E-Cad, C-E-Cad	Knockdown inhibits GC growth and metastasis	GC growth and metastasis regulation	Knockdown studies, Functional
	circ-OXCT1	Inhibits EMT and metastasis in GC	EMT, Metastasis inhibition	Expression analysis, Functional
	circ_0006089	Silencing inhibits GC growth, spread, angiogenesis	GC growth, spread, angiogenesis inhibition	Knockdown studies, Functional
Lung Cancer	hsa_circRNA_0088036	Knocking down reduces NSCLC cell proliferation, invasion	NSCLC progression inhibition	In vitro studies
	circ-WHSC1	Promotes lung cancer cell proliferation, migration	Proliferation, Migration control	In vitro studies
	hsa_circ_0072088, hsa_circ_0008274	Regulation of cancer development; important pathways	EMT, Cancer development regulation	High-throughput data analysis
	cESRP1	Predictive biomarker; changes chemotherapy responsiveness	Chemotherapy responsiveness, TGF-β pathway	Expression analysis, Functional
Hepatocellular Carcinoma	circFGGY	Low expression linked to poor survival; suppresses HCC invasion	Overall survival, Invasion inhibition	Expression analysis, Functional
Oesophageal Squamous Cell Carcinoma	circ-DOCK5	Downregulated in ESCC; mitigates ZEB1-enhanced migration	ZEB1-mediated migration inhibition	Expression analysis, Functional
Bladder Cancer	CircRIP2	Induces EMT; higher expression linked to poor prognosis	EMT, Bladder cancer growth regulation	Expression analysis, Functional
	CircEHBP1	Upregulated in bladder cancer; induces lymphangiogenesis	Lymphangiogenesis, Metastasis induction	Expression analysis, Functional
Prostate Cancer	hsa_circ_0063329	Downregulated; slows PCa cell growth	PCa cell growth regulation	Expression analysis, Functional
	Not specified	IFN-γ induces EMT; hsa_circ_0001165 and hsa_circ_0001085 role	IFN-γ-induced EMT, TNF regulation	High-throughput sequencing, Functional

## 5 Conclusion and future perpspective

In conclusion, this review has explored the important function that Transforming Growth Factor-β (TGF-β) plays in promoting different types of cancer. The investigation of TGF-β′s pro-tumorigenic actions highlights its crucial participation in cancer genesis and progression. TGF-β-activated signalling pathways support important functions such immune evasion, invasion, and cell proliferation, all of which work together to create an environment that is favourable to cancer. The insights gained from understanding TGF-β′s role as a promoter in different cancer types highlight its potential as a target for therapeutic interventions. One strategy to prevent the growth and spread of tumours is to interfere with the TGF-β signalling pathways. Novel approaches to specifically block TGF-β′s pro-tumorigenic actions can be developed as research into the precise pathways promoting carcinogenesis progresses.

Prospects for the future regarding TGF-β and its function as a promoter in different types of cancer are promising in terms of expanding our knowledge and refining treatment approaches. Uncovering the complex signalling cascades that TGF-β particularly contributes to downstream of its pro-tumorigenic actions is one line of investigation. A thorough molecular mapping of these pathways may reveal new targets for targeted therapeutics, enabling the creation of more targeted and potent treatments. Moreover, the discovery of biomarkers linked to tumours caused by TGF-β may provide important resources for early diagnosis and prognosis. Examining the particular genetic and epigenetic changes associated with TGF-β signalling pathway activation might help create diagnostic indicators. Targeted therapy development to interfere with TGF-β′s cancer-promoting function is a viable option in the era of personalized medicine. Treatment alternatives that are less toxic and more precise may be provided by small molecule inhibitors, monoclonal antibodies, or other novel techniques that specifically target essential elements of the TGF-β signalling system. Furthermore, a more comprehensive knowledge of the spatiotemporal dynamics of TGF-β activity within the tumour microenvironment may be obtained by integrating advanced methods like single-cell sequencing and improved imaging modalities. This information can guide approaches to deal with problems like heterogeneity and treatment resistance, eventually increasing the effectiveness of therapeutic interventions.

## Data availability

No data was used for the research described in the article.

## CRediT authorship contribution statement

**Asif Ahmad Bhat:** Formal analysis, Data curation, Conceptualization. **Gaurav Gupta:** Investigation, Formal analysis. **Rajiv Dahiya:** Formal analysis, Conceptualization, Investigation. **Riya Thapa:** Resources, Project administration. **Archana Gahtori:** Supervision, Software. **Moyad Shahwan:** Software, Resources. **Vikas Jakhmola:** Writing – original draft, Visualization. **Abhishek Tiwari:** Writing – review & editing, Investigation. **Mahish Kumar:** Validation, Supervision. **Harish Dureja:** Visualization, Software. **Sachin Kumar Singh:** Resources, Software, Supervision, Validation. **Kamal Dua:** Data curation, Formal analysis, Resources, Visualization. **Vinoth Kumarasamy:** Writing – original draft, Validation. **Vetriselvan Subramaniyan:** Data curation, Conceptualization.

## Declaration of competing interest

The authors declare that they have no known competing financial interests or personal relationships that could have appeared to influence the work reported in this paper.

## References

[1] Mancarella D., Plass C. (2021). Epigenetic signatures in cancer: proper controls, current challenges and the potential for clinical translation. Genome Med..

[2] He A.T. (2021). Targeting circular RNAs as a therapeutic approach: current strategies and challenges. Signal Transduct. Targeted Ther..

[3] Bhat A.A. (2023). A comprehensive review on the emerging role of long non-coding RNAs in the regulation of NF-κB signaling in inflammatory lung diseases. Int. J. Biol. Macromol..

[4] Liu J. (2022). Wnt/β-catenin signalling: function, biological mechanisms, and therapeutic opportunities. Signal Transduct. Targeted Ther..

[5] Yang L. (2021). Functions of circular RNAs in bladder, prostate and renal cell cancer. Mol. Med. Rep..

[6] Lai X.N. (2020). MiRNAs and LncRNAs: dual roles in TGF-β signaling-regulated metastasis in lung cancer. Int. J. Mol. Sci..

[7] Khalilian S. (2023). circGFRA1: a circular RNA with important roles in human carcinogenesis. Pathol. Res. Pract..

[8] Khalilian S. (2023). circWHSC1: a circular RNA piece in the human cancer puzzle. Pathol. Res. Pract..

[9] Liu X. (2022). Circular RNA: an emerging frontier in RNA therapeutic targets, RNA therapeutics, and mRNA vaccines. J. Contr. Release.

[10] Verduci L. (2021). CircRNAs: role in human diseases and potential use as biomarkers. Cell Death Dis..

[11] Almouh M. (2022). Circular RNAs play roles in regulatory networks of cell signaling pathways in human cancers. Life Sci..

[12] Yang Y. (2021). The role of TGF-β signaling pathways in cancer and its potential as a therapeutic target. Evid Based Complement Alternat Med.

[13] Liu S., Ren J., Ten Dijke P. (2021). Targeting TGFβ signal transduction for cancer therapy. Signal Transduct. Targeted Ther..

[14] Tzavlaki K., Moustakas A. (2020). TGF-Β signaling. Biomolecules.

[15] Heldin C.H., Moustakas A. (2016). Signaling receptors for TGF-β family members. Cold Spring Harbor Perspect. Biol..

[16] Liu H., Chen Y.G. (2022). The interplay between TGF-β signaling and cell metabolism. Front. Cell Dev. Biol..

[17] Bai J., Xi Q. (2018). Crosstalk between TGF-β signaling and epigenome. Acta Biochim. Biophys. Sin..

[18] Bhat A.A. (2023). Uncovering the complex role of interferon-gamma in suppressing type 2 immunity to cancer. Cytokine.

[19] Kristensen L.S. (2019). The biogenesis, biology and characterization of circular RNAs. Nat. Rev. Genet..

[20] Rao D. (2021). The emerging roles of circFOXO3 in cancer. Front. Cell Dev. Biol..

[21] Derynck R., Turley S.J., Akhurst R.J. (2021). TGFβ biology in cancer progression and immunotherapy. Nat. Rev. Clin. Oncol..

[22] Garlapati P. (2021). Circular RNAs regulate cancer-related signaling pathways and serve as potential diagnostic biomarkers for human cancers. Cancer Cell Int..

[23] Bhat A.A. (2023).

[24] Batlle E., Massagué J. (2019). Transforming growth factor-β signaling in immunity and cancer. Immunity.

[25] Caja L. (2018). TGF-Β and the tissue microenvironment: relevance in fibrosis and cancer. Int. J. Mol. Sci..

[26] Colak S., Ten Dijke P. (2017). Targeting TGF-β signaling in cancer. Trends Cancer.

[27] Gough N.R., Xiang X., Mishra L. (2021). TGF-Β signaling in liver, Pancreas, and gastrointestinal diseases and cancer. Gastroenterology.

[28] Hao Y., Baker D., Ten Dijke P. (2019). TGF-β-Mediated epithelial-mesenchymal transition and cancer metastasis. Int. J. Mol. Sci..

[29] MaruYama T., Chen W., Shibata H. (2022). TGF-Β and cancer immunotherapy. Biol. Pharm. Bull..

[30] Morikawa M., Derynck R., Miyazono K. (2016). TGF-Β and the TGF-β family: context-dependent roles in cell and tissue physiology. Cold Spring Harbor Perspect. Biol..

[31] Peng D. (2022). Targeting TGF-β signal transduction for fibrosis and cancer therapy. Mol. Cancer.

[32] Wang J. (2021). TGF-beta signaling in cancer radiotherapy. Cytokine.

[33] Zhao M., Mishra L., Deng C.X. (2018). The role of TGF-β/SMAD4 signaling in cancer. Int. J. Biol. Sci..

[34] Huang C.Y. (2021). Recent progress in TGF-β inhibitors for cancer therapy. Biomed. Pharmacother..

[35] Lee J.H., Massagué J. (2022). TGF-β in developmental and fibrogenic EMTs. Semin. Cancer Biol..

[36] Pawlak J.B., Blobe G.C. (2022). TGF-β superfamily co-receptors in cancer. Dev. Dynam..

[37] Farooqi A.A., Naureen H., Attar R. (2022). Regulation of cell signaling pathways by circular RNAs and microRNAs in different cancers: spotlight on Wnt/β-catenin, JAK/STAT, TGF/SMAD, SHH/GLI, NOTCH and Hippo pathways. Semin. Cell Dev. Biol..

[38] Grelet S. (2017). Pleiotropic roles of non-coding RNAs in TGF-β-mediated epithelial-mesenchymal transition and their functions in tumor progression. Cancers.

[39] Liao R. (2021). Current molecular biology and therapeutic strategy status and prospects for circRNAs in HBV-associated hepatocellular carcinoma. Front. Oncol..

[40] Bhat A.A. (2023). The pyroptotic role of Caspase-3/GSDME signalling pathway among various cancer: a Review. Int. J. Biol. Macromol..

[41] Mahmoudian R.A. (2023). Circular RNAs as the pivotal regulators of epithelial-mesenchymal transition in gastrointestinal tumor cells. Pathol. Res. Pract..

[42] Najafi S. (2023). Recent insights into the roles of circular RNAs in human brain development and neurologic diseases. Int. J. Biol. Macromol..

[43] Pan X. (2021). Circular RNAs as potential regulators in bone remodeling: a narrative review. Ann. Transl. Med..

[44] Shang W. (2019). Molecular mechanisms of circular RNAs, transforming growth factor-β, and long noncoding RNAs in hepatocellular carcinoma. Cancer Med..

[45] Shree B., Sharma V. (2023). Role of non-coding RNAs in TGF-β signalling in glioma. Brain Sci..

[46] Bhat A.A. (2023).

[47] Yousefi F. (2020). TGF-β and WNT signaling pathways in cardiac fibrosis: non-coding RNAs come into focus. Cell Commun. Signal..

[48] Capp J.P. (2021). Interplay between genetic, epigenetic, and gene expression variability: considering complexity in evolvability. Evol Appl.

[49] Lin S. (2022). TGF-β1 regulates chondrocyte proliferation and extracellular matrix synthesis via circPhf21a-Vegfa axis in osteoarthritis. Cell Commun. Signal..

[50] Łukasiewicz S. (2021). Breast cancer-epidemiology, risk factors, classification, prognostic markers, and current treatment strategies-an updated review. Cancers.

[51] Fahad Ullah M. (2019). Breast cancer: current perspectives on the disease status. Adv. Exp. Med. Biol..

[52] Thapa R. (2023). Unlocking the potential of mesoporous silica nanoparticles in breast cancer treatment. J. Nanoparticle Res..

[53] Roulot A. (2016). Tumoral heterogeneity of breast cancer. Ann. Biol. Clin..

[54] Chapman N.A., Dupré D.J., Rainey J.K. (2014). The apelin receptor: physiology, pathology, cell signalling, and ligand modulation of a peptide-activated class A GPCR. Biochem. Cell. Biol..

[55] Lin H. (2022). Upregulation of circ_0008812 and circ_0001583 predicts poor prognosis and promotes breast cancer proliferation. Front. Mol. Biosci..

[56] Han J.J., Wang X.Q., Zhang X.A. (2022). Functional interactions between lncRNAs/circRNAs and miRNAs: insights into rheumatoid arthritis. Front. Immunol..

[57] Zhu H. (2023). Dynamic characterization and interpretation for protein-RNA interactions across diverse cellular conditions using HDRNet. Nat. Commun..

[58] Wang T. (2021). Screening and bioinformatics analysis of competitive endogenous RNA regulatory network --Related to circular RNA in breast cancer. BioMed Res. Int..

[59] Baidoun F. (2021). Colorectal cancer epidemiology: recent trends and impact on outcomes. Curr. Drug Targets.

[60] Biller L.H., Schrag D. (2021). Diagnosis and treatment of metastatic colorectal cancer: a review. JAMA.

[61] Li J. (2021). Genetic and biological hallmarks of colorectal cancer. Genes Dev..

[62] Wang H., Tian T., Zhang J. (2021). Tumor-associated macrophages (TAMs) in colorectal cancer (CRC): from mechanism to therapy and prognosis. Int. J. Mol. Sci..

[63] Gupta G. (2023).

[64] Nasri Nasrabadi P. (2022). The pleiotropy of PAX5 gene products and function. Int. J. Mol. Sci..

[65] Medvedovic J. (2011). Pax5: a master regulator of B cell development and leukemogenesis. Adv. Immunol..

[66] Yu J.H. (2023). Hsa_circ_0020134 promotes liver metastasis of colorectal cancer through the miR-183-5p-PFN2-TGF-β/Smad axis. Transl Oncol.

[67] Chao M.V., Rajagopal R., Lee F.S. (2006). Neurotrophin signalling in health and disease. Clin. Sci. (Lond.).

[68] Yang Y. (2020). Whole transcriptome RNA sequencing identified circ_022743, circ_052666, and circ_004452 were associated with colon cancer development. DNA Cell Biol..

[69] Ervin E.H. (2022). Inside the stemness engine: mechanistic links between deregulated transcription factors and stemness in cancer. Semin. Cancer Biol..

[70] Ji S. (2023). Cellular rejuvenation: molecular mechanisms and potential therapeutic interventions for diseases. Signal Transduct. Targeted Ther..

[71] Rengganaten V. (2020). Mapping a circular RNA-microRNA-mRNA-signaling regulatory Axis that modulates stemness properties of cancer stem cell populations in colorectal cancer spheroid cells. Int. J. Mol. Sci..

[72] Vincent A. (2011). Pancreatic cancer. Lancet.

[73] Ilic M., Ilic I. (2016). Epidemiology of pancreatic cancer. World J. Gastroenterol..

[74] Zhao Z., Liu W. (2020). Pancreatic cancer: a review of risk factors, diagnosis, and treatment. Technol. Cancer Res. Treat..

[75] Hussain M.S. (2023). From nature to therapy: luteolin's potential as an immune system modulator in inflammatory disorders. J. Biochem. Mol. Toxicol..

[76] Chen M. (2023). Dysregulation of the circ_0087502/miR-1179/TGFBR2 pathway supports gemcitabine resistance in pancreatic cancer. Cancer Biol. Ther..

[77] Shao T., Pan Y.H., Xiong X.D. (2021). Circular RNA: an important player with multiple facets to regulate its parental gene expression. Mol. Ther. Nucleic Acids.

[78] Das A. (2021). Emerging role of circular RNA-protein interactions. Noncoding RNA.

[79] Zhao Z. (2023). circEIF3I facilitates the recruitment of SMAD3 to early endosomes to promote TGF-β signalling pathway-mediated activation of MMPs in pancreatic cancer. Mol. Cancer.

[80] Correa P. (2013). Gastric cancer: overview. Gastroenterol. Clin. N. Am..

[81] Karimi P. (2014). Gastric cancer: descriptive epidemiology, risk factors, screening, and prevention. Cancer Epidemiol. Biomarkers Prev..

[82] Rao B.V.K. (2023). Morin: a comprehensive review on its versatile biological activity and associated therapeutic potential in treating cancers. Pharmacological Research-Modern Chinese Medicine.

[83] Machlowska J. (2020). Gastric cancer: epidemiology, risk factors, classification, genomic characteristics and treatment strategies. Int. J. Mol. Sci..

[84] Tan Z. (2019). Recent advances in the surgical treatment of advanced gastric cancer: a review. Med Sci Monit.

[85] Li L., Wang X. (2021). Identification of gastric cancer subtypes based on pathway clustering. npj Precis. Oncol..

[86] Liu R. (2020). PI3K/AKT pathway as a key link modulates the multidrug resistance of cancers. Cell Death Dis..

[87] Li F. (2023). Circ-E-Cad encodes a protein that promotes the proliferation and migration of gastric cancer via the TGF-β/Smad/C-E-Cad/PI3K/AKT pathway. Mol. Carcinog..

[88] Mumtaz P.T. (2020). Deep insights in circular RNAs: from biogenesis to therapeutics. Biol. Proced. Online.

[89] Carthew R.W. (2021). Gene regulation and cellular metabolism: an essential partnership. Trends Genet..

[90] Thapa R. (2023). Galangin as an inflammatory response modulator: an updated overview and therapeutic potential. Chem. Biol. Interact..

[91] Liu J. (2020). Circ-OXCT1 suppresses gastric cancer EMT and metastasis by attenuating TGF-β pathway through the circ-OXCT1/miR-136/SMAD4 Axis. OncoTargets Ther..

[92] Chen C. (2021). MicroRNA-3613-3p functions as a tumor suppressor and represents a novel therapeutic target in breast cancer. Breast Cancer Res..

[93] Bradley R.K., Anczuków O. (2023). RNA splicing dysregulation and the hallmarks of cancer. Nat. Rev. Cancer.

[94] Zhou Y. (2022). circ_0006089 promotes gastric cancer growth, metastasis, glycolysis, and angiogenesis by regulating miR-361-3p/TGFB1. Cancer Sci..

[95] Clark J.S. (2022). Post-translational modifications of the p53 protein and the impact in alzheimer's disease: a review of the literature. Front. Aging Neurosci..

[96] Xu M., Liu P.P., Li H. (2019). Innate immune signaling and its role in metabolic and cardiovascular diseases. Physiol. Rev..

[97] Thapa R. (2023). Unveiling the connection: long-chain non-coding RNAs and critical signaling pathways in breast cancer. Pathol. Res. Pract..

[98] Lu R.D. (2022). [Effect of circular RNA hsa_circ_0067582 on the proliferation and invasion ability of gastric cancer cells]. Zhongguo Yi Xue Ke Xue Yuan Xue Bao.

[99] Wang X. (2022). CircCCDC66: emerging roles and potential clinical values in malignant tumors. Front. Oncol..

[100] Tang X. (2021). Review on circular RNAs and new insights into their roles in cancer. Comput. Struct. Biotechnol. J..

[101] Xu G. (2020). Circular RNA CCDC66 promotes gastric cancer progression by regulating c-Myc and TGF-β signaling pathways. J. Cancer.

[102] Hirsch F.R. (2017). Lung cancer: current therapies and new targeted treatments. Lancet.

[103] Nooreldeen R., Bach H. (2021). Current and future development in lung cancer diagnosis. Int. J. Mol. Sci..

[104] Thapa R. (2023). New horizons in lung cancer management through ATR/CHK1 pathway modulation. Future Med. Chem..

[105] Romaszko A.M., Doboszyńska A. (2018). Multiple primary lung cancer: a literature review. Adv. Clin. Exp. Med..

[106] Schabath M.B., Cote M.L. (2019). Cancer progress and priorities: lung cancer. Cancer Epidemiol. Biomarkers Prev..

[107] Bhat A.A. (2022). Polysaccharide-based nanomedicines targeting lung cancer. Pharmaceutics.

[108] Villalobos P., Wistuba I.I. (2017). Lung cancer biomarkers. Hematol. Oncol. Clin. N. Am..

[109] Wang Y. (2020). New insights into small-cell lung cancer development and therapy. Cell Biol. Int..

[110] Bhat A.A. (2022). Nanotechnology-based advancements in NF-κB pathway inhibition for the treatment of inflammatory lung diseases. Nanomedicine (Lond).

[111] Liu H. (2022). Bcl-3: a double-edged sword in immune cells and inflammation. Front. Immunol..

[112] Zhou Y.W. (2021). Therapeutic targets and interventional strategies in COVID-19: mechanisms and clinical studies. Signal Transduct. Targeted Ther..

[113] Ge P. (2023). Hsa_circ_0088036 promotes nonsmall cell lung cancer progression by regulating miR-1343-3p/Bcl-3 axis through TGFβ/Smad3/EMT signaling. Mol. Carcinog..

[114] Baba A.B. (2022). Transforming growth factor-beta (TGF-β) signaling in cancer-A betrayal within. Front. Pharmacol..

[115] Gupta G. (2023). Exploring ACSL4/LPCAT3/ALOX15 and SLC7A11/GPX4/NFE2L2 as potential targets in ferroptosis-based cancer therapy. Future Med. Chem..

[116] Damgaard R.B. (2021). The ubiquitin system: from cell signalling to disease biology and new therapeutic opportunities. Cell Death Differ..

[117] Guan S. (2021). Circular RNA WHSC1 exerts oncogenic properties by regulating miR-7/TAB2 in lung cancer. J. Cell Mol. Med..

[118] Guo Y.J. (2020). ERK/MAPK signalling pathway and tumorigenesis. Exp. Ther. Med..

[119] Lavoie H., Gagnon J., Therrien M. (2020). ERK signalling: a master regulator of cell behaviour, life and fate. Nat. Rev. Mol. Cell Biol..

[120] Santarpia L., Lippman S.M., El-Naggar A.K. (2012). Targeting the MAPK-RAS-RAF signaling pathway in cancer therapy. Expert Opin. Ther. Targets.

[121] Thapa R. (2023). A review of Glycogen Synthase Kinase-3 (GSK3) inhibitors for cancers therapies. Int. J. Biol. Macromol..

[122] Liang L. (2020). Identification of circRNA-miRNA-mRNA networks for exploring the fundamental mechanism in lung adenocarcinoma. OncoTargets Ther..

[123] Wei J. (2023). Understanding the roles and regulation patterns of circRNA on its host gene in tumorigenesis and tumor progression. J. Exp. Clin. Cancer Res..

[124] Statello L. (2021). Gene regulation by long non-coding RNAs and its biological functions. Nat. Rev. Mol. Cell Biol..

[125] Huang W. (2020). Circular RNA cESRP1 sensitises small cell lung cancer cells to chemotherapy by sponging miR-93-5p to inhibit TGF-β signalling. Cell Death Differ..

[126] Forner A., Reig M., Bruix J. (2018). Hepatocellular carcinoma. Lancet.

[127] Ganesan P., Kulik L.M. (2023). Hepatocellular carcinoma: new developments. Clin. Liver Dis..

[128] Gilles H., Garbutt T., Landrum J. (2022). Hepatocellular carcinoma. Crit. Care Nurs. Clin..

[129] Thapa R. (2023). Recent developments in the role of protocatechuic acid in neurodegenerative disorders. EXCLI journal.

[130] Hartke J., Johnson M., Ghabril M. (2017). The diagnosis and treatment of hepatocellular carcinoma. Semin. Diagn. Pathol..

[131] Nagaraju G.P. (2022). Epigenetics in hepatocellular carcinoma. Semin. Cancer Biol..

[132] Ma Y. (2020). Biogenesis and functions of circular RNAs and their role in diseases of the female reproductive system. Reprod. Biol. Endocrinol..

[133] Misir S., Wu N., Yang B.B. (2022). Specific expression and functions of circular RNAs. Cell Death Differ..

[134] Yu C.Y., Kuo H.C. (2019). The emerging roles and functions of circular RNAs and their generation. J. Biomed. Sci..

[135] Feng K.L. (2022). CircFGGY inhibits cell growth, invasion and epithelial-mesenchymal transition of hepatocellular carcinoma via regulating the miR-545-3p/smad7 Axis. Front. Cell Dev. Biol..

[136] Abnet C.C., Arnold M., Wei W.Q. (2018). Epidemiology of esophageal squamous cell carcinoma. Gastroenterology.

[137] Baba Y. (2020). Tumor immune microenvironment and immune checkpoint inhibitors in esophageal squamous cell carcinoma. Cancer Sci..

[138] Codipilly D.C., Wang K.K. (2022). Squamous cell carcinoma of the esophagus. Gastroenterol. Clin. N. Am..

[139] Thapa R. (2022). Current update on the protective effect of epicatechin in neurodegenerative diseases. EXCLI journal.

[140] Lee N.P. (2018). Tumor xenograft animal models for esophageal squamous cell carcinoma. J. Biomed. Sci..

[141] Morgan E. (2022). The global landscape of esophageal squamous cell carcinoma and esophageal adenocarcinoma incidence and mortality in 2020 and projections to 2040: new estimates from GLOBOCAN 2020. Gastroenterology.

[142] Pennathur A. (2013). Oesophageal carcinoma. Lancet.

[143] Greene J. (2017). Circular RNAs: biogenesis, function and role in human diseases. Front. Mol. Biosci..

[144] Kukimoto-Niino M. (2021). Cryo-EM structure of the human ELMO1-DOCK5-Rac1 complex. Sci. Adv..

[145] Meng L. (2021). ZEB1 represses biogenesis of circ-DOCK5 to facilitate metastasis in esophageal squamous cell carcinoma via a positive feedback loop with TGF-β. Cancer Lett..

[146] Ahmadi H., Duddalwar V., Daneshmand S. (2021). Diagnosis and staging of bladder cancer. Hematol. Oncol. Clin. N. Am..

[147] Compérat E. (2022). Current best practice for bladder cancer: a narrative review of diagnostics and treatments. Lancet.

[148] Bhat A.A. (2023). Neuropharmacological effect of risperidone: from chemistry to medicine. Chem. Biol. Interact..

[149] Dobruch J., Oszczudłowski M. (2021). Bladder cancer: current challenges and future directions. Medicina (Kaunas).

[150] Kirkali Z. (2005). Bladder cancer: epidemiology, staging and grading, and diagnosis. Urology.

[151] Martinez Rodriguez R.H., Buisan Rueda O., Ibarz L. (2017). Bladder cancer: present and future. Med. Clin..

[152] Chen L.-L. (2020). The expanding regulatory mechanisms and cellular functions of circular RNAs. Nat. Rev. Mol. Cell Biol..

[153] Sharma A.R. (2021). Recent research progress on circular RNAs: biogenesis, properties, functions, and therapeutic potential. Mol. Ther. Nucleic Acids.

[154] Su Y. (2020). circRIP2 accelerates bladder cancer progression via miR-1305/Tgf-β2/smad3 pathway. Mol. Cancer.

[155] Zhou W.-Y. (2020). Circular RNA: metabolism, functions and interactions with proteins. Mol. Cancer.

[156] Yu F., He H., Zhou Y. (2023). Roles, biological functions, and clinical significances of RHPN1-AS1 in cancer. Pathol. Res. Pract..

[157] Zhu J. (2021). circEHBP1 promotes lymphangiogenesis and lymphatic metastasis of bladder cancer via miR-130a-3p/TGFβR1/VEGF-D signaling. Mol. Ther..

[158] Li K. (2020). ILF3 is a substrate of SPOP for regulating serine biosynthesis in colorectal cancer. Cell Res..

[159] Huang Y. (2022). RNA binding protein POP7 regulates ILF3 mRNA stability and expression to promote breast cancer progression. Cancer Sci..

[160] Li W., Deng X., Chen J. (2022). RNA-binding proteins in regulating mRNA stability and translation: roles and mechanisms in cancer. Semin. Cancer Biol..

[161] Li P. (2023). Characterization of circSCL38A1 as a novel oncogene in bladder cancer via targeting ILF3/TGF-β2 signaling axis. Cell Death Dis..

[162] Achard V. (2022). Metastatic prostate cancer: treatment options. Oncology.

[163] Grozescu T., Popa F. (2017). Prostate cancer between prognosis and adequate/proper therapy. J Med Life.

[164] Nguyen-Nielsen M., Borre M. (2016). Diagnostic and therapeutic strategies for prostate cancer. Semin. Nucl. Med..

[165] Sekhoacha M. (2022). Prostate cancer review: genetics, diagnosis, treatment options, and alternative approaches. Molecules.

[166] Wang G. (2018). Genetics and biology of prostate cancer. Genes Dev..

[167] Lv D. (2023). Hsa_circ_0063329 inhibits prostate cancer growth and metastasis by modulating the miR-605-5p/tgif2 axis. Cell Cycle.

[168] Pestka S., Krause C.D., Walter M.R. (2004). Interferons, interferon-like cytokines, and their receptors. Immunol. Rev..

[169] Walter M.R. (2020). The role of structure in the biology of interferon signaling. Front. Immunol..

[170] Yan Z. (2020). Screening and identification of epithelial-to-mesenchymal transition-related circRNA and miRNA in prostate cancer. Pathol. Res. Pract..

